# Systemic Lupus Erythematosus complicated by Neuromyelitis Optica (Devic’s Syndrome): case series from a single paediatric rheumatology centre

**DOI:** 10.1186/1546-0096-9-S1-P241

**Published:** 2011-09-14

**Authors:** Despoina Maritsi, Muthana Al-Obadi, Sonia Melo-Gomes, Kjell Tullus, Clarissa A Pilkington

**Affiliations:** 1 Department of Paediatric and Adolescent Rheumatology, Great Ormond Street Hospital for Children NHS Trust, London, UK; 2 Department of Paediatric Nephrology, Great Ormond Street Hospital for Children NHS Trust, London, UK

## Objective

To identify the incidence of Neuromyelitis Optica (NMO) in patients with paediatric Systemic Lupus Erythematosus (SLE) and describe its pattern of presentation.

## Background

NMO or Devic’s syndrome is a rare autoimmune demyelinating disease of the central nervous system manifesting with transverse meylitis involving three or more continuous segments and optic neuritis in the presence of NMO IgG antibodies.

## Methods

Retrospective case study of all SLE patients with CNS symptoms, diagnosed from 2000-2010 and review of the clinical data, laboratory and MRI findings. Data were collected from the medical records and were analyzed using SPSS 2010.

Setting: A tertiary referral centre for juvenile SLE.

## Results

A total of 210 (161 females, 49 males) SLE patients were indentified, 39 of which had manifestations of potential CNS involvement and underwent imaging of their CNS including spinal cord. Three were indentified with probable Devic syndrome, which was confirmed in two (0.9%). Both patients were adolescent females and of Caucasian origin. In one patient NMO was the first manifestation of SLE. In the other NMO developed three years following diagnosis of SLE. They both presented with deterioration of visual acuity, localized spine tenderness and malaise. NMO was confirmed based on MRI findings and the presence of raised Aquaporin-4 IgG antibodies in the plasma. On both occasions NMO had a relapsing course and interestingly NMO relapses coincided with SLE disease flare-up, which responded to treatment simultaneously. The patient with ongoing SLE had a more severe course and required more intensive immunosuppressive treatment. Both patients developed depression following diagnosis of NMO.

## Discussion

SLE is a multisystemic autoimmune disease and 25% of the patients will develop CNS involvement throughout its course. While NMO has been described in adult patients with SLE, these cases derive specifically from a paediatric population. We believe that SLE and NMO are parts of the same disease spectrum. When this condition is noticed in patients with refractory, long standing SLE, prognosis is guarded. The burden of chronic illness on mental health status and on deterioration of quality of life is well documented in adults. However, physicians working with paediatric patients should be reminded of the psychological component that needs to be addressed in each visit.

